# Years of Life Lost Due to Cervical Cancer in Poland in 2000 to 2015

**DOI:** 10.3390/ijerph16091545

**Published:** 2019-05-01

**Authors:** Małgorzata Pikala, Monika Burzyńska, Irena Maniecka-Bryła

**Affiliations:** Department of Epidemiology and Biostatistics, the Chair of Social and Preventive Medicine of the Medical University of Lodz, 90-136 Lodz, Poland; monika.burzynska@umed.lodz.pl (M.B.); irena.maniecka-bryla@umed.lodz.pl (I.M.-B.)

**Keywords:** cervical cancer, neoplasms, years of life lost, mortality, epidemiology, trends, socioeconomic factors, Poland

## Abstract

The aim of the study was an analysis of years of life lost due to cervical cancer in Poland in the period 2000 to 2015 with consideration given to differences related to education and place of residence. The study material was 28,274 death certificates of all female inhabitants of Poland, who died in 2000 to 2015 due to cervical cancer. In order to calculate years of life lost, the authors used indices: SEYLL_p_ (Standard Expected Years of Life Lost per living person), SEYLL_d_ (per deaths) and AAPC (Average Annual Percentage Change). The SEYLL_p_ index (per 100,000) due to cervical cancer in Poland decreased from 394.3 in 2000 to 220.9 years of life in 2015 (AAPC = −3.6%). Women with university education lost the smallest number of years of life (SEYLL_p_ = 139.0 in 2000 and 53.7 in 2015; AAPC = −5.4%), whereas those with elementary education had the greatest number of years of life lost (524.2 and 312.8; AAPC = −3.4%). Women living in rural areas lost on average 329.5 years in 2000 and 177.0 in 2015 (AAPC = −3.8%). In city areas, the values were 428.6 and 247.1 (AAPC = −3.4%). Many of the years of life lost could have been avoided by including more women, particularly those with elementary education, in screening examinations.

## 1. Introduction

Cervical cancer is the fourth most common neoplasm affecting female population in the world and the seventh most common cancer in the general population worldwide [1]. In Europe, it occupies the fourth and eighth place, respectively [2,3]. New cases constitute, on average, 8.3% of all neoplasms diagnosed in females all over the world and 6.6% of those diagnosed in European countries [1]. In 2013, 485,000 new cases of cervical cancer were diagnosed worldwide, and 236,000 deaths were caused by this disease, which contributed to 6.9 million disability adjusted life-years (DALY) in the female population. Standardized age-adjusted incidence rate, calculated per 100,000 females was 15.7 in developing and 9.9 in developed countries, whereas the mortality rate due to this cause was 8.3 and 3.3, respectively [2]. In 1990, cervical cancer was the eighth most common factor of all kinds of neoplasm which contributed to prematurely lost years of life. In the period between 1990 and 2013 the incidence and mortality rates due to cervical cancer decreased. This trend, however, did not cause major changes because at that time, the disease became the ninth most common neoplasm, contributing to lost years of life [4].

According to statistics of the Agency for Research on Cancer, Poland is a country with a high incidence of cervical cancer and a great number of cervical cancer-related deaths. Each year, about 3000 new cases of cervical cancer are detected. In 2015, the age-standardized death rate (SDR) due to this cause was 7.9 per 100,000 females. Neoplasm of the cervix is not the most common disease. However, it is closely monitored due to the fact that it still poses a serious threat both in Poland and in other European countries. Since we are knowledgeable of the etiology of the disease and are able to diagnose it in its early stage and reduce it (by vaccination), we can prevent it. [5]. Results of the Polish study seem to be alarming. They indicate that of all European countries, Poland is characterized with the lowest percentage of 5-year survival of cervical cancer patients because this rate in patients aged 15 to 99 years in Poland is 56.4% [6] and the mean value for European countries is 62.4% [7].

Cervical cancer is not only an important health issue but also a social one because it affects young women (aged 35 to 59 years) who are professionally active and raising children. It is also important from an economic point of view because both direct costs, including diagnostics, treatment and prophylaxis, and indirect costs, generated by premature mortality and disabilities which affect patients are high [8,9].

Measures expressing premature mortality are more and more commonly applied in epidemiological studies. They are expressed in units of lost years of life and enable the evaluation of the social and economic consequences of premature mortality [10,11,12,13,14,15]. Moreover, the application of such indices of years of life lost is helpful in evaluating a treatment course which involves decreasing the number of deaths and extending patients’ survival.

The aim of the study was an analysis of mortality trends and the number of years of life lost due to cervical cancer in Poland in the period 2000 to 2015 with consideration given to differences related to education and place of residence (urban area–rural area).

## 2. Materials and Methods

The study material was a database including 5,996,489 death certificates of all inhabitants of Poland who died in the period 2000 to 2015. Of this number, 28,274 women died of cervical cancer (according to the International Statistical Classification of Diseases and Health Related Problems—Tenth Revision—ICD-10 coded as C53). The data were provided by the Department of Information of the Polish Central Statistical Office. The procedure for coding causes of death in Poland is performed in a similar manner to those carried out in the majority of countries in the world, by focusing on the so called ‘primary cause of death’, or the disease which triggered a pathological process, leading to death.

The authors calculated crude deaths rates (CDR) and SDR.

$$CDR=\frac{k}{p}*100,000$$

where: *k*—number of cervical cancer deaths; *p*—number of women above the age of 15 years.

The standardization procedure was performed with the use of direct method, in compliance with the European Standard Population, updated in 2012 [16].

$$SDR=\frac{\sum_{i=1}^{N}\frac{ki}{pi}wi}{\sum_{i=1}^{N}wi}$$

where: *k_i_* is the number of cervical cancer deaths in this *i*-age group, *p_i_* is population size of this *i*-age group, *w_i_* is the weight assigned to this *i*-age group, resulting from the distribution of the standard population, *N*—number of the age groups

Years of life lost were calculated and analyzed by the method described in Global Burden of Disease (GBD) [17]. The SEYLL index (Standard Expected Years of Life Lost) was used to calculate the number of years of life lost by the studied population in comparison to the years lost by the referential (standard) population.

There are various methods for calculating years of life lost, and the main difference between them is a point of reference, i.e., the level of mortality which is considered ‘ideal’. In the Global Burden of Disease (GBD) study 2010, WHO experts recommend using life tables, based on the lowest noted death rate for each age group, in countries with population above five million [18].

In this study, the SEYLL index was calculated according to the following formula:

$$SEYLL=\overset{l}{\underset{x=0}{\sum }}d_{x}e_{x}^{*}$$

where: $e_{x}^{*}$—life expectancy, based on GBD 2010 life tables, *d_x_*—number of deaths at age *x*, *x*—age at which the person died, *l*—last age which the population reaches.

The authors also applied the SEYLL per person (SEYLL_p_) index, which is a ratio of SEYLL and the size of the population, calculated per 100,000 inhabitants, and the SEYLL per death (SEYLL_d_) index, being a ratio of SEYLL and the number of deaths due to a particular cause i.e., it expresses the number of YLL calculated per one dead person [19]. The analysis of time trends has been carried out with joinpoint models and Joinpoint Regression program, a statistical software package developed by the U.S. National Cancer Institute for the Surveillance, Epidemiology and End Results Program [20]. This method is an advanced version of linear regression, where the time trend is expressed with a broken line, which is a sequence of segments joined in joinpoints. In these points, the change of the value is statistically significant (*p* < 0.05). We have also calculated annual percentage change (APC) for each segment of broken lines and average annual percentage change (AAPC) for the whole study period with corresponding 95% confidence intervals (CI).

In order to compare the number of years of life lost by women due to cervical cancer in particular categories, i.e., education level, place of residence (city area/rural area), the authors calculated SEYLL_p_ Ratio (SR), which is a ratio of SEYLL_p_ in less privileged groups and SEYLL_p_ in more privileged groups with corresponding 95% confidence intervals (CI) [21].

## 3. Results

The number of deaths due to cervical cancer in Poland in the 21st century is gradually decreasing. In the year 2000, 1987 women died of this disease and the CDR was 10.1 per 100,000 females aged 15 years or older (Table 1). In 2015, the number of deaths was 1,585, whereas CDR was 8.0 per 100,000 women. APC in 2000 to 2015 was −1.2% (Table 2). SDR were calculated so as to eliminate the impact of the changing age of women in Poland. In 2000, the value of SDR was 12.0, but in 2015, it decreased to 7.9 per 100,000 females. APC of SDR was faster than for CDR and its value was −2.4%.

**Figure 1:**
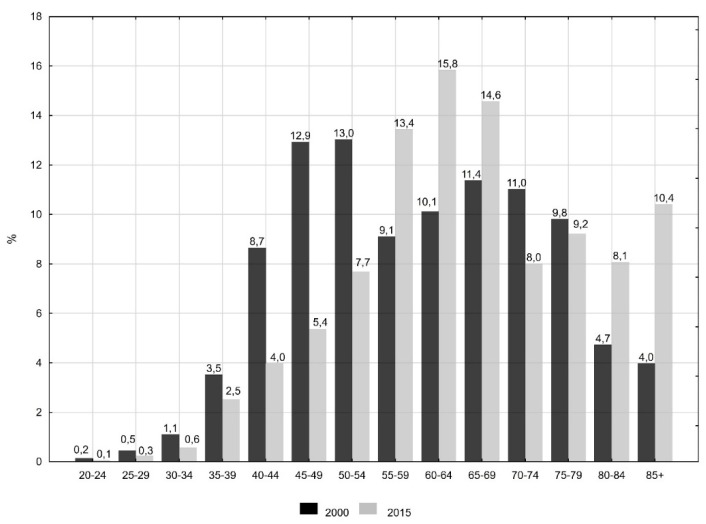
Percentage of deaths due to cervical cancer by age groups in the years 2000 and 2015 in Poland.

In 2000, the highest percentage of deaths due to cervical cancer was observed for women aged 45 to 54 years, but in 2015, the women were aged 60 to 64 years (Figure 1). Despite these positive trends, the percentage of premature deaths (before the age of 65 years) was still very high and in 2015, it was 49.7%, which contributed to a great number of lost years of women’s life.

In 2000, deaths due to cervical cancer contributed to a loss of 55,730 SEYLL, which corresponded to 394.3 years per 100,000 females (Table 1). In 2015, the absolute number of years of life lost was 37,634 years and this corresponded to 220.9 years of life per 100,000 females. In 2000to 2015, Average AAPC was −3.6%; between 2000 and 2002, the decrease was more rapid and its value was −9.9% and after the year 2002, it decreased to −2.6% per annum (Table 2).

Each woman who died of cervical cancer in Poland in 2000 lost on average 28.1 years of life; in 2015, the SEYLL_d_ value decreased to 23.7 years (Table 1). AAPC was −1.2% for the whole study period and in 2000 to 2002, APC was −0.8%, whereas after 2002, it was −2.6%.

**Figure 2:**
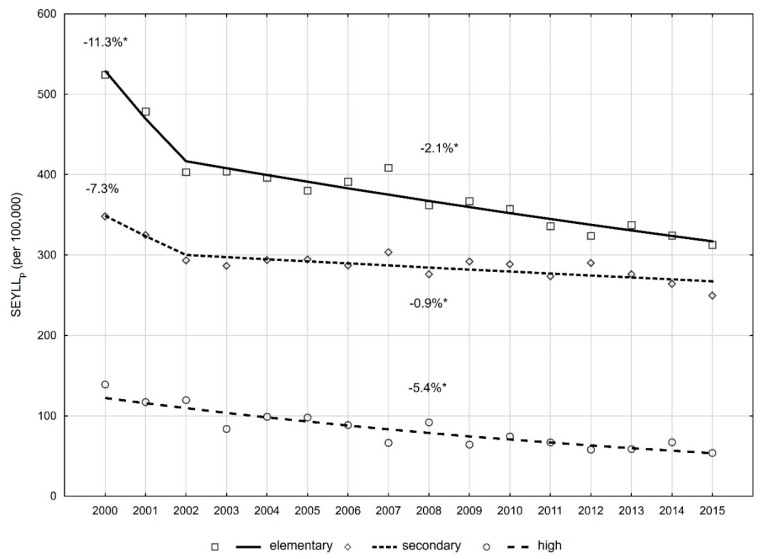
Time trends of the SEYLL_p_ index by education in 2000 to 2015 in Poland.

In order to check a relationship between education level and the rate of mortality due to cervical cancer, the authors calculated the SEYLL_p_ index for three different education levels of women, aged 15 years or older in Poland: Elementary, secondary, and high. Women with high education lost the smallest number of years of life. In 2000, SEYLL_p_ was 139.0, whereas in 2015, its value decreased to 53.7 per 100,000 (Table 3). In women with secondary education, SEYLL_p_ was 348.1 in 2000 and 249.6 in 2015. In regard to women with elementary education, the values were 524.2 and 312.8, respectively per 100,000. The lowest SEYLL_p_ values in the group of women with high education corresponded to average annual percentage change equal to −5.4% (Figure 2). In the group of women with secondary education, AAPC was −1.8% and −3.4% in the group of women with elementary education (Table 2). Consequently, disproportions regarding the number of years of life lost due to cervical cancer, related to education level, are increasing. SR, indicating a ratio of SEYLL_p_ in women with secondary education to SEYLL_p_ in women with university education was 2.5 in 2000 and 4.6 in 2015. In 2000, women with elementary education lost 3.8 times more years of life than those with university education. In 2015, SR was 5.8.

**Figure 3:**
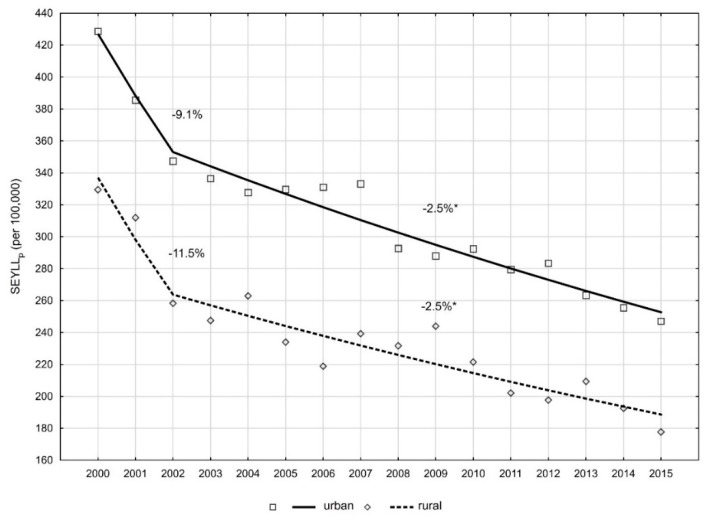
Time trends of the SEYLLp index by place of residence in 2000 to 2015 in Poland.

Slightly smaller, however also statistically significant, differences were observed for the number of years of life lost by women for different places of residence. Women inhabiting rural areas lost on average 329.5 years in 2000 and 177.0 in 2015 as calculated per 100,000. For female inhabitants of the city, the values were 428.6 and 247.1, respectively (Table 3). AAPC for SEYLL_p_ of countrywide dwellers was −3.8% and was slightly quicker than in the city (−3.4%) (Table 2). In 2000 to 2002, a slightly quicker pace of decrease was observed for inhabitants of rural areas than for inhabitants of city areas (−11.5% vs. −9.1%). After 2002, the decline pace in the city was the same as the one noted for the countrywide and its value was −2.5% (Figure 3). SR for the city and countryside was 1.3 in 2000 and 1.4 in 2015.

## 4. Discussion

The results of our study indicate that the number of years of life lost due to cervical cancer in Poland is gradually decreasing. This decline is caused by the decrease in the incidence rates (from 19.0 per 100,000 in 2000 to 13.7 in 2015) and mortality rates (from 10.1 per 100,000 in 2000 to 8.0 in 2015).

The incidence of cervical cancer is influenced by the exposure to etiological/risk-modifying factors. Exposure to genital human papilloma virus (HPV) infection is an obligatory (but not sufficient) risk factor of cervical cancer [22]. The risk increases with an earlier age of sexual debut, the number of lifetime sexual partners and births, and with the use of oral contraceptives and smoking [23,24]. Condom use decreases the risk of cervical cancer [25]. Decline of incidence rates in Poland may be associated with declining number of births over recent decades, and the rise in condom use among young women noted between 1991 and 2004/2005 and generally declining rates of smoking among young women [26]. The decrease in mortality is probably the result of the earlier detection of cervical cancer by screening tests and improvement in the effectiveness of treatment. Between 2000 and 2010 the survival rate improved for patients with cervical cancer by 2.7 percentage points (53.7% vs 56.4%) [6].

Cervical cancer is the fourth most common cause of death contributing to the highest average number of years of life lost by one woman who died in Poland [27]. The authors of this study confirmed that the value of the index decreased from 28.1 years in 2000 to 23.7 in 2015.

Despite this positive trend, the mortality rate due to this cause is still much higher than in western countries of the European Union. The lowest cervical cancer-related mortality and incidence rates are observed in countries which implemented national programmes of active prophylaxis (Finland, Sweden, Holland). Within oncological prophylaxis, Poland implemented the National Programme for Control of Cancerous Diseases in 2005, which also included the National Population-Based Cervical Cancer Screening Programme. In 2010, this programme received the Pearl of Wisdom Award from the European Cervical Cancer Association. Unfortunately, the percentage of Polish participants in these screening examinations is not satisfactory. In Finland, it is 67.4%, in Holland—66.3%, in Denmark—64.4%, but in Poland in 2015 it was only 18.2% [28].

Despite low response-rate in screening tests, the positive effects of an organized programme that utilizes three-yearly Pap smears, seem to be noticeable. Accelerations of downward trends in cervical cancer incidence and mortality are most apparent in women within the age limits of the organized screening programme (25 to 59 years). In contrast, the downward trend in cervical cancer incidence and mortality in women aged 60+ was stopped around 2005 [26].

Health inequalities, related to socioeconomic status, particularly education, is a crucial issue, and this poses a challenge in attempts to control cervical cancer. Many studies indicate that the level of education is the most important indicator of socioeconomic status. Other social parameters, such as profession and income account for differences in education levels [29]. A number of studies indicate that low socioeconomic status is an important factor determining the incidence of cervical cancer [30,31,32]. The authors of this study noted that in 2015 women with elementary education lost 5.8 times years more than women with university education. In 2000, SR was 3.8, which means that education-related disproportions are increasing.

Malignant neoplasms pose a more serious life threat for inhabitants of cities rather than for those living in rural areas [33]. The authors observed that this refers also to neoplasms of the cervix, since in the year 2015, women living in the city lost 1.4 years more than women living in the countryside.

Obligatory vaccination against HPV might, to some extent, decrease cervical cancer-related incidence and mortality. In countries in which vaccination against HPV is commonly applied, the number of cases of cervical cancer decreased by half [34]. Unfortunately, vaccines against HPV are currently only recommended in Poland but they are not financed by the Ministry of Health. Local authorities in some towns, gminas, and poviats decided to cover the cost of administration of HPV vaccines.

## 5. Limitations

The quality of the analyses performed on the mortality statistics depend on the completeness and accuracy of the information contained in the death certificate and the proper and precise description of the cause of death. Poland is a country with 100% completeness of death registration. In order to standardize death causes, which are subject to further statistical analyses, it was determined that the doctor who pronounces the death is responsible for filling in the death card, into which he or she puts the primary, secondary, and direct cause of death, whereas qualified teams of doctors are responsible for coding death causes according to the ICD-10 classification.

The data relating to 2012 shows that the cause of more than 28% of deaths (about 109.000) were inaccurately described. However, in the majority of cases (78,500) it concerned deaths due to cardiovascular diseases [35].

## 6. Conclusions

Despite decreasing mortality trends, neoplasms of the cervix are still a serious problem for public health in Poland. The number of years of life lost points out the social and economic aspect of a loss related to premature mortality due to cervical cancer in Poland. Further monitoring of cervical cancer-related incidence and mortality trends, as well as implementation of multidirectional strategies that would expand knowledge of risk factors are definitely required. Such awareness would lead to a greater need of undergoing screening examinations, particularly in the group of women characterized with lower socioeconomic status.

## Figures and Tables

**Table 1:** Number of deaths and values of crude death rate (CDR), standardized death rates (SDR), standard expected years of life lost (SEYLL), standard expected years of life lost per living person (SEYLL_p_), and standard expected years of life lost per death (SEYLL_d_) due to cervical cancer in Poland in 2000 to 2015.

Year	Number of Deaths	CDR (per 100,000)	SDR (per 100,000)	SEYLL	SEYLL_p_ (per 100,000)	SEYLL_d_ (per Deaths)
2000	1987	10.1	12.0	55,730	394.3	28.1
2001	1826	9.3	10.8	51,128	360.0	28.0
2002	1855	9.4	10.9	51,721	315.5	27.9
2003	1825	9.3	10.6	50,228	304.5	27.5
2004	1819	9.3	10.3	50,501	304.3	27.8
2005	1796	9.1	10.0	49,219	295.0	27.4
2006	1824	9.3	10.1	48,671	290.3	26.7
2007	1907	9.7	10.5	50,294	298.9	26.4
2008	1745	8.8	9.5	45,622	270.3	26.1
2009	1748	8.9	9.4	45,993	271.8	26.3
2010	1735	8.7	9.1	45,310	266.2	26.1
2011	1656	8.3	8.7	42,743	250.7	25.8
2012	1669	8.4	8.6	42,891	251.4	25.7
2013	1669	8.4	8.5	41,455	243.1	24.8
2014	1628	8.2	8.2	39,518	231.7	24.3
2015	1585	8.0	7.9	37,634	220.9	23.7

**Table 2:** Time trends of CDR, SDR, SEYLL_p_, and SEYLL_d_ due to cervical cancer in Poland in 2000 to 2015—joinpoint regression analysis.

Coefficients	Number of Joinpoints	Years	APC (95% CI)	AAPC (95% CI)
CDR	0	2000–2015	−1.2 * (−8.4; −0.9)	
SDR	0	2000–2015	−2.4 * (−2.7; −2.1)	
SEYLL_p_	1	2000–2002	−9.9 * (−16.7; −2.6)	−3.6 * (−4.5; −2.6)
		2002–2015	−2.6 * (−3.0; −2.2)	
SEYLL_d_	1	2000–2002	−0.8 * (−1.0; −0,7)	−1.2 * (−1.4; −0.9)
		2002–2015	−2.6 * (−3.9; −1.2)	
SEYLL_p_ according to level of education				
high	0	2000–2015	−5.4 * (−6.7; −4.0)	
secondary	1	2000–2002	−7.3 (−17.7; 4.5)	−1.8 * (−3.2; −0.3)
		2002–2015	−0.9 * (−1.5; −0.3)	
elementary	1	2000–2002	−11.3 * (−20.1; −1.5)	−3.4 * (−4.6; −2.1)
		2002–2015	−2.1 * (−2.6; −1.5)	
SEYLL_p_ according to place of residence				
urban	1	2000–2002	−9.1 (−17.9; 0.6)	−3.4 * (−4.7; −2.2)
		2002–2015	−2.5 * (−3.1; −2.0)	
rural	1	2000–2002	−11.5 (−25.6; 5.2)	−3.8 * (−5.9; −1.7)
		2002–2015	−2.5 * (−3.4; −1.7)	

* *p* < 0.05.

**Table 3:** SEYLL_p_ and SEYLL_p_ Ratio (SR) by level of education and place of residence, 2000 and 2015, Poland.

Risk Factors	SEYLL_p_		SR (95% CI)	
	2000	2015	2000	2015
**Educational level**				
**high (ref)**	139.0	53.7	1.0	1.0
**secondary**	348.1	249.6	2.5 (2.4; 2.6)	4.6 (4.4; 4.8)
**elementary**	524.2	312.8	3.8 (3.6; 4.0)	5.8 (5.5; 6.1)
**Place of residence**				
**rural (ref)**	329.5	177.7	1.0	1.0
**urban**	428.6	247.1	1.3 (1.2; 1.3)	1.4. (1.3; 1.4)
